# Supplementary material for: Patterns of Intron Gain and Loss in Fungi
Source: PLoS Biol. 2004 Nov 30;2(12):e422. doi: 10.1371/journal.pbio.0020422 (PMC532390; doi:10.1371/journal.pbio.0020422)
Supplement: Table S1 — Also available at http://genes.mit.edu/NielsenEtAl/. (4.3 MB ZIP). [file pbio.0020422.st001.zip › NielsenEtAl/html/634.html]

AN6114.1.NCU01321.1.MG06908.1.FG01896.1


```
 CLUSTAL W (1.82) Multiple Sequence Alignments - Introns Inserted


Sequence 1: NCU01321.1	602 aa
Sequence 2: MG06908.1	582 aa
Sequence 3: FG01896.1	594 aa
Sequence 4: AN6114.1	665 aa
Alignment Length: 692 aa
Number Identitical Residues: 189 aa
Alignment Score (without introns) 10207


MG06908.1 	------------------------------MALH-FPVIYLLASHMDPDELYSIEEQIP-
NCU01321.1	------------------------------MTLD-FPVIFLLPTNLGADELHELEEQIP-
FG01896.1 	------------------------------MSLTNLPRIFLLSTKLKPEELHQLEERIP-
AN6114.1  	MSSTSTASLTPSDPGQDLDIEADLLTTESGFSLAKCPPIYVLPTHLDLDALHQTEETLIQ
          	 ::::::: :.:..... . .:.  ::.:.::* . * *::*.:::  : *:. ** : .

MG06908.1 	---TLTYDVREADVVIGKVGKRERALFELRRIGLKTSEVPLGN-LEANEPPTKRRRLP--
NCU01321.1	---TLTYDINEAEVILGNVHRKQRVLFELRKHKLVTEEISTTETDEPTSPSPKRRRFQTP
FG01896.1 	---TLTYDIGEAEIVIGKISQPMRAEFELRRAKLNFVPLSQAQTRRDRPSSTAVTKDP--
AN6114.1  	HGARLTYDVAEATLILGKVRQKKRAALELRSRGVWTEEVESTSSHTSGPPAKRRRLESGR
          	 .: ****: ** :::*:: :  *. :***   :    :   .:     ..         

MG06908.1 	------------EHDATQD----------PEAKMGDTTTPTTVGSL-------IKVAKLA
NCU01321.1	GS------VITEDSDTASDGD------FHPLLTQSTQSTLKATPTN-------VKVVKLA
FG01896.1 	--------------SNGSD----------PKRRRVTGNTTGGHDT--------VKVVKLE
AN6114.1  	NGSAVSEIYLSTEDESEEDGRRLTAHLEHPQLSESKRSERTSVSKLLSKDEGVIRVVRLD
          	..:: :.   : . .  .*.   ::    *       .      .  :.... ::*.:* 

MG06908.1 	WFTDSIAKGEVLPIDDYVVYQG-------INLGREEPFPPIN---------QIAASFKEG
NCU01321.1	WFTESLKAGTVLPIQDYTLYRGRKQQSKPAKPQQASPTKAAN---------IIKRALADP
FG01896.1 	WLLDSWEKENMLPIEQYLVCQVSKVSAREATPVPVPVLGSESEHMLSAGSTSPASGILER
AN6114.1  	WLAQCAEAQTLLPLASFVVYHAR----KIERPATRGDDETAN-----------SQAILDR
          	*: :.     :**: .: : :                  . .             .: : 

MG06908.1 	AAAKQAG-TGSAHSMPSFKRRRGQPQSY---VPHLMPESTSEHDAAENLPPIPSFLHS--
NCU01321.1	KPSSHGSRPGSSHSTTSHGRR-DHFITK---APALLHMTTSEHEV--ELPPIPAYLHT--
FG01896.1 	ALAEQKDKPTSTSPQTRHKRRHDGSTTLSQNAPSLLRQTTSEHDI--VLPTMPEFLRT--
AN6114.1  	AKQDAAFKAPFPPSTDKHDRVASHRAPP-----KLYRQTTSENEEAEPLPPPPEWVRDGV
          	   .    .  . .   . *  .   .       *   :***:: :. **. * ::: . 

MG06908.1 	VYSCQRPTPANPPNSAFIEQLKEIRTTRLLNGDSIGVRAYSTAIASLAAYPYGLTTHIE1
NCU01321.1	TYACQRPAPTHPPNEPFIEELMKIRTARTLLGDKIGVRAYSSAIAALISYPYPLQSAFE~
FG01896.1 	AYSCERPTYKNPPNKAFVKLLTEIRTIRQLGEDDVGVRAYSTSIASVAAYPYPLKNSQE1
AN6114.1  	MYACMRSSFLNTPNNAFINQLVKIRTIRELNLDEIGVRAYSTAIAAIAAYPYEIRRGSQ~
          	 *:* *.:  :.**..*:: * :*** * *  *.:******::**:: :*** :    : 

MG06908.1 	VQRLPGCNEKIAFLYQEWKHT--GKIKEVEEATQDPRLKILREFHATWGVGDTTAREWYN
NCU01321.1	VARLPGCGLKIAQLYQEFREQ--GELQEAKEDESDPRLAVLKLFHEIWGVAETTAREFYN
FG01896.1 	VARLPGCGDRIAELWHRWKET--GESAEVREANADPRISVLKIFYDIWGVGAATAREFYQ
AN6114.1  	ILSLPGCDSKIANLFVEFKASESGTLAAAEALDKDPDLRTLHTFYNIWGVGSKTAREFFY
          	:  ****. :** *: .::  .:*    ..    ** :  *: *:  ***.  ****:: 

MG06908.1 	-KG~WRDLDDVVEYGWESLTRVQQIGVKYYDELQLKISREEVEKIANIILQHAN--KIRS
NCU01321.1	-KG~WRDLDDIVEYGWDTLTRVQQIGVKYYDEFQQKIARAEVESIANIILEHAN--KIHP
FG01896.1 	-KG1WRDLVDLVEHGWNTLSRSQQIGVKYYDEFLSGIPRDEVASIGAAILKHAR--IIDP
AN6114.1  	ARQ~WRDLDDIVEHGWHSLSRVQQIGVKYYDEFLLDIPREETEAIGKIICQHANSASVRP
          	::  **** *:**:**.:*:* **********:   *.* *.  *.  * :**.:: : .

MG06908.1 	-------GFQMVIVGGYRRGKDESGDVDLILSHPDESATDRFVTMLVDSLSDSDYVTHTL
NCU01321.1	-------GFQMVIVGGYRRGKLASGDVDVVLSHPDKAATRGFVEQIVVALEQSNYITHTL
FG01896.1 	-------GFEMVIVGGHRRGKQKSGDVDVVLSHRDESKTLHVISKIVVILEQAHLVTHTL
AN6114.1  	TAQYDGHGVECIIVGSYRRGKPVSHDVDIILSHRDEAITHNLVVDVVASLESEGWITHTL
          	::. .. *.: :***.:****  * ***::*** *:: *  .:  :*  *..   :****

MG06908.1 	SYTVKNSERGQKPVAWKGSAPKPHGSGFDTLDKALVVWQEP-------KPGAKKNPNPHR
NCU01321.1	ILSTANTERGQEPVAWKGNEKK-SGTGFDTLDKALVVWQDPH-----WTTDEKKNPIPHR
FG01896.1 	VLSAHNSERGQRPVSWKGQTSS--SSGFDTLDKALVVWKDP-----------SKEDAPHR
AN6114.1  	ALHLTTSKRDQQTLPYRGDDDD--RNHFDSLDKALVVWQTPKDKLGSGTQASSANTNPHR
          	     .::*.*..:.::*.  .   . **:********: * .. .: .   . :  ***

MG06908.1 	QVDIIISPWKTAGCAVLGWTSGTTFQRDMRKYCKEKLNLKFDSSGIRSRADG-RWVDLES
NCU01321.1	RVDIIISPWKTAGCAVLGWTSGTTFQRDLRRYCKKERSLKFDSSGIRSRADG-TWVDFES
FG01896.1 	RVDIIISPWKTVGCAVLGWSGGTTFQRDLRQYCKYVKGYKFDSSGIRHRADG-RWVDLEG
AN6114.1  	RVDIIVSPWRTVGCAVLGWSGDTTFERDLRRYAKKAHGWKFDSSGVRERVTGGQVIDLEA
          	:****:***:*.*******:..***:**:*:*.*   . ******:* *. *.  :*:*.

MG06908.1 	R-DGVPAPDMHTAERRVFEGLKLEWRPPTERNTG
NCU01321.1	GPNGEPAPDMLTAERRVFEGLGLEWRPPEERCTG
FG01896.1 	TSADNAAPDMETAERRVFGGLGLPWRPPEARCTG
AN6114.1  	R-----GETWQERERMVMEGLGVGWRPPEERCTR
          	      .      ** *: ** : ****  * *
```
